# Cardiac Extracellular Vesicles (EVs) Released in the Presence or Absence of Inflammatory Cues Support Angiogenesis in Different Manners

**DOI:** 10.3390/ijms20246363

**Published:** 2019-12-17

**Authors:** Christien Madlen Beez, Maria Schneider, Marion Haag, Kathleen Pappritz, Sophie Van Linthout, Michael Sittinger, Martina Seifert

**Affiliations:** 1 Charité-Universitätsmedizin Berlin, BCRT-Berlin Institute of Health (BIH) Center for Regenerative Therapies, 10178 Berlin, Germany; 2 Institute of Medical Immunology, Charité-Universitätsmedizin Berlin, Corporate Member of Freie Universität Berlin, Humboldt-Universität zu Berlin, and Berlin Institute of Health, 13353 Berlin, Germany; 3 Tissue Engineering Laboratory, Charité-Universitätsmedizin Berlin, Corporate Member of Freie Universität Berlin, Humboldt-Universität zu Berlin, and Berlin Institute of Health, 10117 Berlin, Germany; 4 Department of Internal Medicine and Cardiology, Campus Virchow Klinikum, Charité-Universitätsmedizin Berlin, Corporate Member of Freie Universität Berlin, Humboldt-Universität zu Berlin, and Berlin Institute of Health, 13353 Berlin, Germany; 5 DZHK (German Center for Cardiovascular Research), Partner Site, 10115 Berlin, Germany

**Keywords:** extracellular vesicles, angiogenesis, VEGF, regeneration, cardiac therapy, intracellular uptake

## Abstract

Cells release extracellular vesicles (EVs) to communicate in a paracrine manner with other cells, and thereby influence processes, such as angiogenesis. The conditioned medium of human cardiac-derived adherent proliferating (CardAP) cells was recently shown to enhance angiogenesis. To elucidate whether their released EVs are involved, we isolated them by differential centrifugation from the conditioned medium derived either in the presence or absence of a pro-inflammatory cytokine cocktail. Murine recipient cells internalized CardAP-EVs as determined by an intracellular detection of human proteins, such as CD63, by a novel flow cytometry method for studying EV–cell interaction. Moreover, endothelial cells treated for 24 h with either unstimulated or cytokine stimulated CardAP-EVs exhibited a higher tube formation capability on Matrigel. Interestingly, unstimulated CardAP-EVs caused endothelial cells to release significantly more vascular endothelial growth factor and interleukin (IL)-6, while cytokine stimulated CardAP-EVs significantly enhanced the release of IL-6 and IL-8. By nCounter^®^ miRNA expression assay (NanoString Technologies) we identified microRNA 302d-3p to be enhanced in unstimulated CardAP-EVs compared to their cytokine stimulated counterparts, which was verified by quantitative polymerase chain reaction. This study demonstrates that both CardAP-EVs are pro-angiogenic by inducing different factors from endothelial cells. This would allow to select potent targets for a safe and efficient therapeutic application.

## 1. Introduction

Cell-cell communication between neighboring and distant cells is crucial for the survival of an organism. It initiates essential processes, such as growth, differentiation or defense mechanisms against pathogens. A direct communication between neighboring cells is ensured by structural connections between them, like tight junctions, while it is enabled in an indirect manner by releasing signaling molecules into the extracellular space [1,2,3,4,5]. Those molecules can be used by the cell itself (autocrine) or by cells in their more immediate vicinity as well as over greater distances (paracrine or endocrine for hormones). Knowledge about mechanisms triggered by this cellular crosstalk can help to improve or even to evolve new therapeutic approaches. For example, processes such as angiogenesis, are highly desired to be enhanced for treating diseases, like those of the cardiovascular system [6,7]. In contrast, a rogue communication can affect the homeostasis of an organism and manifest diseases. Malignant degenerated cells, for example, have altered their communication to facilitate the formation of metastases and to evade immune responses [8,9].

In the last decades, extracellular vesicles (EVs) have been identified as a major paracrine mediator. They contain a variety of signaling molecules, including diverse RNA types, such as messenger RNA or micro RNA (miRNA), receptors, enzymes, and lipids. Three different types of EVs can be distinguished by their biogenesis and diameter. Exosomes, the smallest subtype (diameter < 0.1 µm), are derived intracellularly in so called multivesicular bodies, while microvesicles (diameter range = 1–0.1 µm) bud directly from the cell membrane and apoptotic bodies (diameter > 1 µm) are formed by the disassembly of apoptotic cells [10,11,12,13,14,15]. Recent in vitro and in vivo studies demonstrated that beneficial therapeutic effects were initiated by EVs originating from regenerative cells of diverse sources [16,17,18,19,20]. A commonly observed phenomenon was the pro-angiogenic effect of EVs derived from mesenchymal stromal cells (MSCs) [21,22], endothelial cells [23,24], as well as cardiac derived cells [17,19,25]. However, not only EVs from “therapeutic” cell sources but also EVs from cancer cells are able to enhance angiogenesis [26].

The potential mechanisms of action and molecules delivered by EVs that affect the vascular development, growth and maturation have been recently reviewed [27]. Here, a wide spectrum of molecules contained within EVs was described, ranging from growth factors, like vascular endothelial growth factor (VEGF), to miRNAs, such as miRNA 126. Since the cellular milieu during the biogenesis of EVs crucially affects their cargo composition, their biological effects can change subsequently. MSC-EVs derived under limited oxygen concentration (hypoxia) showed superior pro-angiogenic features compared to their counterparts derived under normoxia conditions [28,29,30,31]. Another important environmental parameter is inflammation, which can be mimicked in the cell culture by supplementing the media with pro-inflammatory cytokines. Endothelial cells treated with interleukin 3 (IL-3) released EVs with superior pro-angiogenic capacity compared to their unstimulated counterpart as shown by Lombardo et al. [23]. Several studies have predominantly investigated the role of cytokine stimulated EVs derived from mesenchymal cells in the context of immunomodulation [32,33]. However, it is not yet completely understood how an inflammatory microenvironment influences the angiogenic features of mesenchymal or cardiac cell derived EVs [29,34]. To try and understand this, we isolated EVs from the conditioned medium of human cardiac-derived adherent proliferating (CardAP) cells that were cultured either in the presence or absence of a pro-inflammatory cytokine cocktail containing IL-1 beta (IL-1β), interferon gamma (IFN-γ), and the tumor necrosis factor alpha (TNF-α). CardAP cells were chosen as source of EVs, because the conditioned medium of this MSC-like cell type already demonstrated to enhance tube formation capabilities of human umbilical vein endothelial cells (HUVECs) [35]. Furthermore, CardAP cells displayed a set of other cardioprotective effects, including immunomodulation and the improvement of heart functions in virus or angiotensin II induced cardiovascular disease models [36,37,38,39]. Since we were already able to show that the immune modulating effect of CardAP cells is partially facilitated by their released EVs [40], it would be of further interest if the cardioprotective feature of enhancing angiogenesis is also facilitated by CardAP-EVs. This knowledge would be useful for a future therapeutic cell-free approach for the treatment of cardiovascular diseases by using CardAP-EVs. Therefore, we not only focused on the impact of unstimulated and cytokine stimulated CardAP-EVs on HUVECs but also on their interaction with possible cardiovascular target cells to evaluate them further as a tool for treating heart diseases.

In the present study, unstimulated as well as cytokine stimulated CardAP-EVs demonstrated to enhance the tube formation capabilities of HUVECs. Interestingly, although both CardAP-EVs showed the same beneficial effect, the pro-angiogenic molecules released from HUVECs were different. HUVECs treated with unstimulated CardAP-EVs released significantly more VEGF and IL-6, whereas cytokine stimulated CardAP-EVs enhanced the release of IL-6 and IL-8 from HUVECs. Our results indicate that this difference was due to an altered miRNA composition between unstimulated and cytokine stimulated CardAP-EVs. Indeed, a potent VEGF inducing miRNA, namely miRNA302d-3p, was found to be enriched in unstimulated CardAP-EVs in comparison to their cytokine stimulated counterparts. Furthermore, we introduced a novel method to demonstrate the intracellular uptake of CardAP-EVs by cardiac murine cells, which would additionally support the miRNA transfer into recipient cells.

Overall, our results support that the inflammatory microenvironment during EV biogenesis influences the content composition of CardAP-EVs and thereby mediate their general pro-angiogenic feature via different pathways. Furthermore, the pro-angiogenic effect of CardAP-EVs would argue for their suitability as a future therapeutic application to treat cardiovascular diseases.

## 2. Results

### 2.1. CardAP-EVs are Interacting with Cardiac Murine Cells

**Figure 1:**
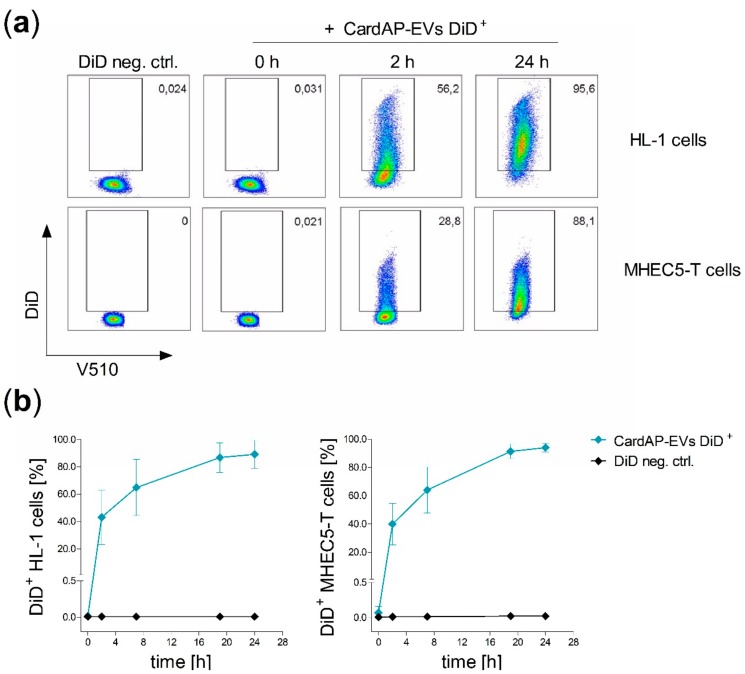
CardAP-EVs (EVs from cardiac derived adherent proliferating cells) interact equally with cardiomyocytes and cardiac endothelial cells. DiD labelled CardAP-EVs (CardAP-EVs DiD^+^) as well as a DiD negative control (DiD neg. ctrl.) were applied to cultured HL-1 cells or MHEC5-T cells (2 × 10^5^ cells/well). At different time points (0, 2, 7, 19, and 24 h) cells were washed twice with PBS, harvested and labelled with a dead/viable marker (V510) for 20 min and fixed with 0.5% PFA after a washing step. All samples were analyzed by flow cytometry at the Canto II. (**a**): Representative dot plots are shown for different time points of HL-1 cells (upper raw) as well as for MHEC5-T cells (lower raw) treated with CardAP-EVs or treated for 24 h with the control. (**b**): The frequency of DiD^+^ HL-1 cells (right, *n* = 4, 4 different CardAP donors) and DiD^+^ MHEC5-T cells (left, *n* = 4–6, 3 different CardAP donors) is shown in relation to the time of incubation (h) as median with interquartile range for treatment with either CardAP-EVs (blue) or control (black).

The size, phenotype and immune modulating capacity of CardAP-EVs has been recently determined [40]. In this study, we focused on the interaction between CardAP-EVs and cardiovascular target cells to determine their impact on angiogenesis as a means to further evaluate their potential as a tool for treating heart diseases. First, CardAP-EVs were isolated by differential centrifugation from the conditioned medium from CardAP cells cultured either in the presence or in the absence of a pro-inflammatory cytokine cocktail containing 10 ng/mL of IFNγ, TNFα, and IL-1β. Then, CardAP-EVs were investigated for their ability to interact with cardiac endothelial cells in comparison to cardiomyocytes using well-characterized murine cells lines. To visualize the interaction, CardAP-EVs were fluorescently labelled with DiD or PKH26. DiD labelled CardAP-EVs (CardAP-EVs DiD^+^) were applied for 24 h either to cultured cardiomyocytes (HL-1 cells) or cardiac endothelial cells (MHEC5-T cells). Both cell types were additionally treated with a DiD labelled unconditioned medium, which served as control (DiD neg. ctrl.) to record background signals. Analysis by flow cytometry revealed that the frequency of DiD^+^ HL-1 cells as well as DiD^+^ MHEC5-T cells (Figure 1a,b) increased over the time period, while the DiD negative controls had very low or undetectable levels of DiD. After 24 h, nearly all HL-1 cells (mean = 89% DiD^+^ HL-1 cells) as well as MHEC5-T cells (mean = 94% DiD^+^ MHEC5-T cells) displayed a DiD^+^ signal indicating an uptake of DiD^+^ CardAP-EVs. Additionally, the interaction was visualized by fluorescence microscopy. Here, HL-1 cells were labelled with DiD, while CardAP-EVs and the negative control were labelled with PKH26. In accordance with the flow cytometry results, an increase in the CardAP-EVs PKH26^+^ signal was detected over the time period of 24 h (Figure S1). After 24 h, some HL-1 cells showed an accumulation of separate single PKH26^+^ signals for CardAP-EVs in the proximity of their nucleus, while other cells displayed no signal (Figure S1).

### 2.2. CardAP-EVs are Predominantly Internalized by Cardiomyocytes and Cardiac Vascular Cells

**Figure 2:**
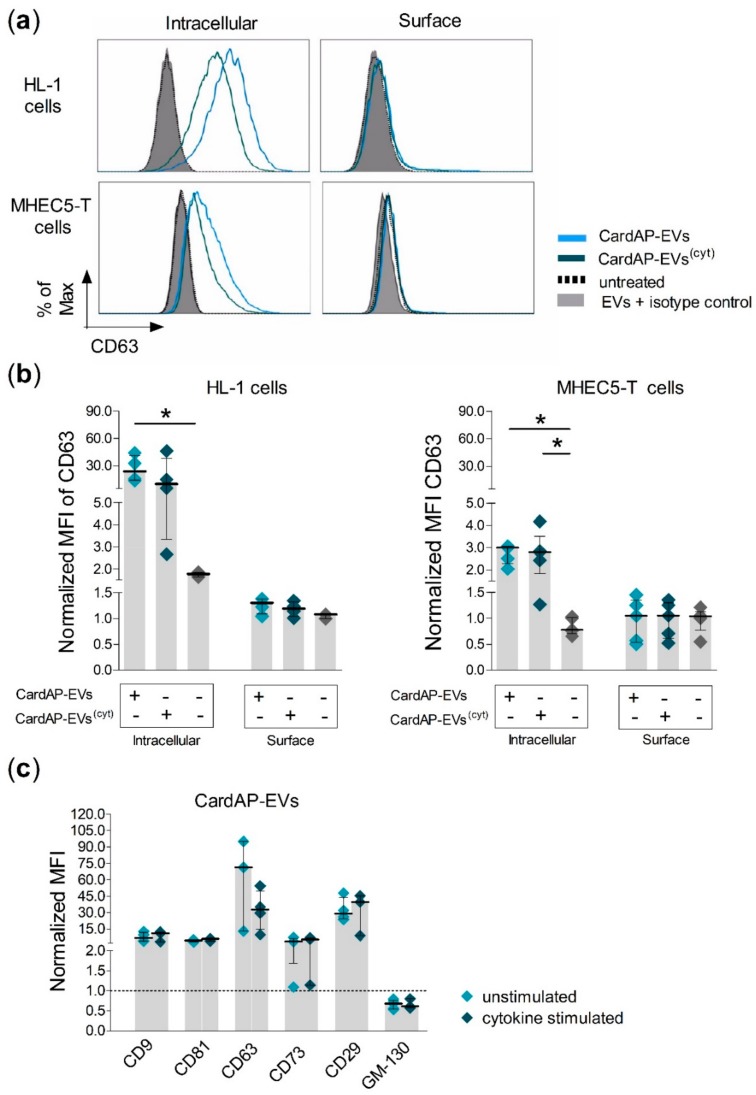
CardAP-EVs are internalized by cardiac murine cells. Human CardAP-EVs either unstimulated (CardAP-EVs) or cytokine stimulated (CardAP-EVs^(cyt)^) were applied to cultured murine HL-1 cells or MHEC5-T cells (2 × 10^5^ cells/well). Additionally, an untreated murine cell control was included. After 24 h, these cells were harvested and stained with anti-human fluorescently labelled antibodies for CD63, using either an intracellular or a cell surface staining protocol. Stained samples were measured by flow cytometry at the Canto II. (**a**): Representative histograms are shown for HL-1 cells (upper row) and MHEC5-T cells (lower row) for human CD63 via intracellular (left) or surface (right) staining in comparison to untreated and isotype control (EVs + isotype ctrl.) (**b**): The mean fluorescent intensity (MFI) for human CD63 was normalized to an unstained control (intracellular or surface staining) and the normalized MFI is shown as median with interquartile range for both staining conditions as well as different treatments for HL-1 cells (left, *n* = 4, 3 different CardAP donors) and MHEC5-T cells (right, *n* = 5, 3 different CardAP donors). (**c**): The presence of human markers (CD9, CD81, CD63, CD73, CD29) and the Golgi matrix protein (GM-130) on CardAP-EVs was verified by flow cytometry of EVs bound to sulfate/aldehyde beads. The normalized MFI are shown for all markers as median with interquartile range (*n* = 3–4, 3 different CardAP donors). Statistical analysis was performed by Kruskal-Wallis test (with Dunn’s post hoc test; * *p* < 0.05).

In a next step, it was analyzed in more detail how CardAP-EVs interact with cells. Several possibilities can be envisioned, including an intracellular uptake of the EVs, binding of the EVs to the cell surface or even a fusion of the EVs with the plasma membrane of the cell. The results from the microscopic evaluation indicated that CardAP-EVs are taken up intracellularly by HL-1 cells. To determine if CardAP-EVs were in fact intracellular, we implemented a novel method in EV research to discriminate between intracellular and surface bound CardAP-EVs. Here, murine cells (HL-1 and MHEC5-T) were treated for 24 h with CardAP-EVs. Since our isolated CardAP-EVs are derived from a human source, antibodies against human proteins would only bind proteins of CardAP-EV origin. Flow cytometry was used to detect whether EVs were localized either intracellularly or on the surface of murine cells by two parallel performed staining protocols. When murine cells were treated for 24 h with either unstimulated (CardAP-EVs) or cytokine stimulated (CardAP-EVs^(cyt)^) CardAP-EVs, the human tetraspanin CD63 was predominantly detected rather intracellularly than on the cell surface (Figure 2a,b). Likewise, other human proteins, like another tetraspanin (CD81) as well as 5’-nucletoidase (CD73), were also found to be mainly intracellularly rather than extracellularly localized (Figure S2). Importantly, no unspecific antibody binding was detected for untreated murine cells or for the isotype control staining (Figure 2a,b and Figure S2). Additionally, the presence of these and other human markers, like integrin β-1 (CD29) and another tetraspanin (CD9), was verified for CardAP-EVs themselves by flow cytometry (Figure 2c). A Golgi matrix protein (golgin subfamily A member 2; GM-130) was incorporated into the analysis to rule out possible cell organelle contaminations in the CardAP-EV samples. GM130 was not detectable in neither unstimulated nor cytokine stimulated CardAP-EV preparations (Figure 2c), arguing against any contamination by cell organelles. Contrarily, GM130 was determined in the apoptotic body fraction collected during EV isolation, which served as positive control for the presence of organelles (Figure S2).

### 2.3. Unstimulated and Cytokine Stimulated CardAP-EVs Enhance Angiogenesis of HUVECs by Altering Their Release of Different Pro-Angiogenic Cues

**Figure 3:**
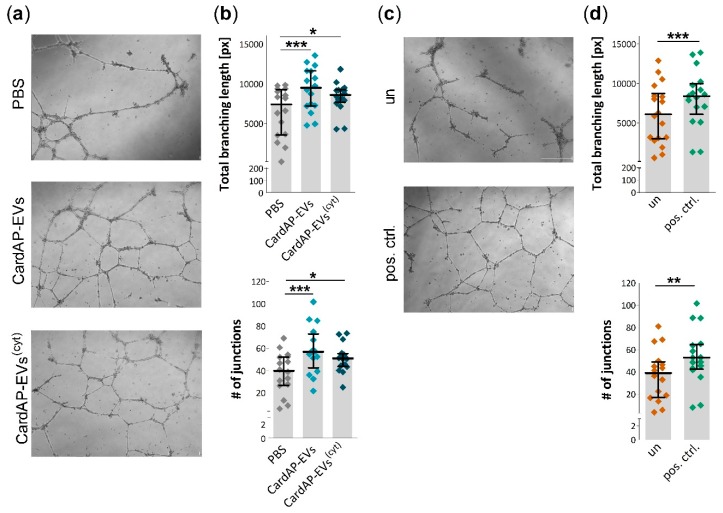
Tube formation capabilities of HUVECs is enhanced by both unstimulated and cytokine stimulated CardAP-EVs. 24 h prior to an assay, HUVECs were treated with 6 µg/mL of either unstimulated (CardAP-EVs) or cytokine stimulated CardAP-EVs (CardAP-EVs^(cyt)^), PBS in corresponding volumes of CardAP-EVs, or left untreated (un). The next day, HUVECs were harvested and applied to a Matrigel-coated well for 20 h. As a positive control, HUVECs were treated in parallel with 10 ng/mL VEGF. The tube formation was documented by light microscopy and pictures were analyzed with the help of the ImageJ Angiogenesis Plugin. (**a**): Representative pictures are shown for HUVECs treated with PBS, unstimulated and cytokine stimulated CardAP-EVs with a scale bar representing 500 µm. (**b**): The quantitative analysis of the tube formation shows a significant increase of the total branching length (upper graph) and the number of junctions (lower graph) for unstimulated as well as cytokine stimulated CardAP-EVs. Individual data points are shown and summarized as median with interquartile range (*n* = 16, 6 different CardAP donors). (**c**): Representative pictures are shown for HUVECs without any treatment (un) or for HUVECs treated with 10 ng/mL VEGF (pos. ctrl.). The scale bar represents 500 µm. (**d**): The total branching length (upper graph) as well as the number of junctions (lower graph) is significantly increased by the application of VEGF versus the untreated samples. Individual data points are shown and summarized as median with interquartile range (*n* = 17). Statistical analysis for three groups was performed by repeated measures ANOVA (Bonferroni post hoc test, *** *p* < 0.001, * *p* < 0.05). Statistical analysis of two groups was performed by paired T test (*** *p* < 0.001, ** *p* < 0.01).

It was already shown that the conditioned medium of CardAP cells increases the tube formation capabilities of HUVECs [35]. To investigate if CardAP-EVs are playing a role in this paracrine mediated pro-angiogenic effect, a Matrigel-based tube formation assay was performed. HUVECs were treated 24 h prior to the assay with either unstimulated or cytokine stimulated CardAP-EVs, PBS as EV vehicle control or they were left untreated. The next day, the pre-treated HUVECs were seeded to Matrigel-coated wells and observed for the next 20 h by light microscopy. Treatment with either unstimulated or cytokine stimulated CardAP-EVs enhanced the tube formation capabilities of HUVECs as shown in representative images (Figure 3a). The total tube length and the number of junctions was significantly increased in HUVECs treated with either unstimulated or cytokine stimulated CardAP-EVs in comparison to the PBS control (Figure 3b). Interestingly, unstimulated CardAP-EVs showed a trend towards a greater pro-angiogenic effect than their cytokine stimulated counterparts. Furthermore, a positive control (HUVECs treated with pro-angiogenic VEGF) significantly increased the total tube length as well as the number of junctions in comparison to untreated HUVECs (Figure 3c,d).

**Figure 4:**
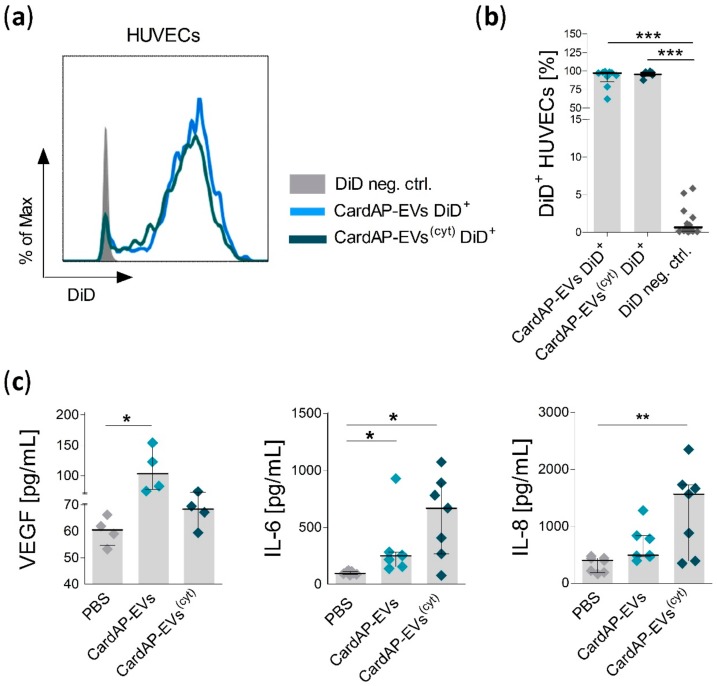
The interaction of unstimulated and cytokine stimulated CardAP-EVs with HUVECs triggers the release of different pro-angiogenic factors. Cultured HUVECs (1.9 × 10^5^ cells/well) were treated with DiD labelled CardAP-EVs or with a DiD negative control. After 24 h, HUVECs were washed twice with PBS, harvested, and labelled with a dead/viable marker (V510) for 20 min and fixed with 0.5% PFA after a washing step. All samples were analyzed by flow cytometry at the Canto II. (**a**): Representative histograms are shown for HUVECs treated with the DiD negative control (DiD neg. ctrl.), DiD labelled unstimulated CardAP-EVs (CardAP-EVs DiD^+^) or cytokine stimulated CardAP-EVs (CardAP-EVs^(cyt)^ DiD^+^) (**b**): The frequency of DiD^+^ HUVECs (*n* = 6–13, 4 different CardAP donors) is shown as median with interquartile range for the three different treatments. (**c**): HUVECs were treated with 6 µg/mL of either unstimulated (EVs), cytokine stimulated CardAP-EVs (EVs^(cyt)^) or PBS in volumes corresponding to that of the CardAP-EVs. The next day, HUVECs were washed twice and fresh medium was applied for 24 h. Then, the medium was collected for detecting IL-6, IL-8, and VEGF by ELISA. The cell correction factor was determined by crystal violet staining. The cytokine concentrations in relation to the cell numbers are shown for VEGF (*n* = 4, 4 different CardAP donors), IL-6, and IL-8 (both *n* = 7, 4 different CardAP donors) as median with interquartile range. Statistical analysis was performed by Friedman test (with Dunn’s post hoc test; *** *p* < 0.001, ** *p* < 0.01, * *p* < 0.05).

A 24-h pre-incubation was performed prior to a Matrigel-based tube formation assay to allow time for interactions to occur between HUVECs and CardAP-EVs. Indeed, the interaction was verified by analysis of HUVECs that incubated with DiD labelled CardAP-EVs (median ≥ 96.7% DiD^+^ HUVECs, Figure 4a,b). To evaluate pro-angiogenic factors released by HUVECs, we washed and applied fresh medium to those pre-incubated HUVECs for another 24 h. Interestingly, HUVECs changed the released amount of pro-angiogenic factors due to treatment with either unstimulated or cytokine stimulated CardAP-EVs. Unstimulated CardAP-EVs significantly increased the release of VEGF and IL-6 in comparison to PBS-treated HUVECs, whereas cytokine stimulated CardAP-EVs did not affect VEGF, but significantly increased the secretion of IL-6 and IL-8 from HUVECs (Figure 4c).

### 2.4. Unstimulated and Cytokine Stimulated CardAP-EVs Differ in Their miRNA Profile

**Figure 5:**
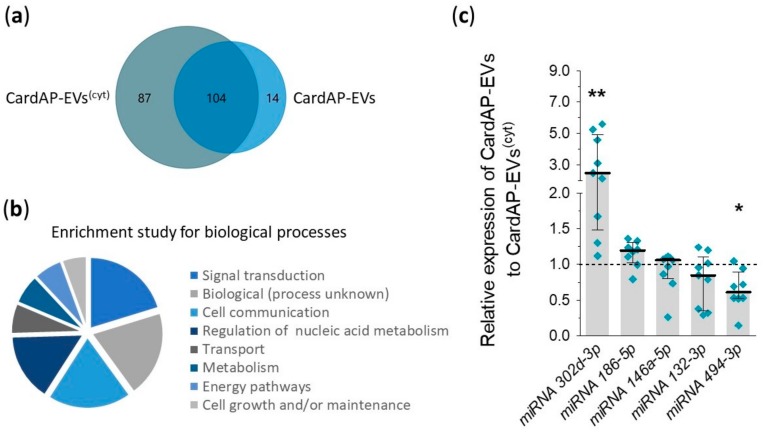
Different composition of miRNAs between unstimulated and cytokine stimulated CardAP-EVs. RNA was isolated from unstimulated and cytokine stimulated CardAP-EVs and used for nCounter^®^ miRNA expression assay as stated in the materials and methods section. (**a**,**b**): The derived data was normalized and used for further analysis by FunRich Analysis for a comparison of observed miRNAs between both conditions illustrated as Venn diagram (**a**) or as general analysis of observed miRNAs for their involvement in biological processes as displayed as pie chart for the top eight processes (**b**). (**c**): Five miRNAs (miR302d, miR186-5p, miR146a-5p, miR132-3p, and miR494-3p) identified in the global nCounter^®^ miRNA expression analysis, were validated by qPCR. Values of unstimulated CardAP-EVs were normalized to the cytokine stimulated CardAP-EVs (set to 1, black dotted line) by means of ΔΔCt analysis. The relative expression is shown as median with interquartile range (*n* = 8–9, 3 different CardAP donors). Statistical analysis was performed with a Wilcoxon signed rank test (** *p* < 0.01, * *p*< 0.05).

The miRNA profile of unstimulated and cytokine stimulated CardAP-EVs from three different CardAP donors was accessed by nCounter^®^ Human v3 miRNA expression assay (NanoString Technologies) to better understand the differently induced pro-angiogenic factors. This assay allows one to determine the expression level of nearly 800 human miRNAs. After background correction and median normalization, a total of 205 miRNAs were detected in CardAP-EVs, 104 miRNAs were found in both CardAP-EV populations, whilst 87 miRNAs were found exclusively in cytokine stimulated CardAP-EVs and 14 miRNAs exclusively in unstimulated CardAP-EVs (Figure 5a). Further examination of all miRNAs by FunRich analysis revealed that they are involved in processes of signal transduction, cell communication or unknown biological processes (top three, Figure 5b). Five miRNAs, listed in Table 1, were selected due to their role in angiogenesis and verified by qPCR analysis. The relative expression for miRNA 302d-3p was significantly enhanced in unstimulated CardAP-EVs related to the cytokine stimulated counterparts. This verifies the results of the nCounter^®^ miRNA expression assay results, where miRNA 302d-3p was found to be the most enriched miRNA in unstimulated CardAP-EVs in comparison to the cytokine stimulated CardAP-EVs. In contrast, the relative expression level for miRNA 494-3p was significantly diminished in unstimulated CardAP-EVs, which indicated an enhanced expression in the cytokine stimulated CardAP-EVs (Figure 5c). Surprisingly, miRNA 494-3p was also detected in unstimulated CardAP-EVs by qPCR, since this miRNA was determined by nCounter^®^ miRNA expression assay solely in the cytokine stimulated CardAP-EVs.

## 3. Discussion

There is a need to identify mechanisms by which EVs modulate angiogenesis to either optimize their features promoting tissue regeneration or to evolve strategies limiting their pathological role in tumor development. In order to harness their therapeutic potential, it is important to understand how EVs interact with endothelial cells and what they carry to regulate angiogenesis. Since the function of EVs can be strongly influenced by the microenvironment they experience during their biogenesis [32,47], we modulated the milieu of well-characterized regenerative cardiac cells to gather insights into how those changes might affect the pro-angiogenic features of their released EVs. For this purpose, CardAP cells were cultured in the presence or absence of pro-inflammatory cytokines. The medium obtained under both conditions was used to isolate CardAP-EVs, which were then compared for their impact on cytokine or growth factor secretion and tube formation capabilities of endothelial cells.

Isolated CardAP-EVs from both unstimulated and cytokine stimulated sources acted as potent inducers of angiogenesis as observed by the significantly enhanced number of junctions and total branching length of HUVECs in Matrigel-based tube formation assays. Additionally, we observed an increased release of pro-angiogenic factors by HUVECs after their treatment with unstimulated or cytokine stimulated CardAP-EVs. Surprisingly, this pro-angiogenic effect was induced by the release of different pro-angiogenic cytokines. Unstimulated CardAP-EVs mediated a significant increased release of VEGF and IL-6 by HUVECs, whereas cytokine stimulated CardAP-EVs triggered a significant enhanced release of IL-6 and IL-8. All three factors have already been described as pro-angiogenic inducers [48,49] as well as being involved in the pro-angiogenic features of EVs [27]. Yet, it was not shown that differently stimulated EVs, either from cardiac cells or from MSCs, caused those different release profiles of pro-angiogenic factors by HUVECs. As other factors were not investigated in this study, it cannot be ruled out that CardAP-EVs also affect other mediators of angiogenesis, like the epidermal growth factor or the platelet-derived growth factor, which could contribute to an enhanced tube formation capability as well [27,50].

In our previous investigation of unstimulated and cytokine stimulated CardAP-EVs, some proteins transported by CardAP-EVs were already identified to be involved in angiogenesis [40]. One such protein identified by mass spectrometry was galectin-1, which was observed on both unstimulated and cytokine stimulated CardAP-EVs. This carbohydrate-binding protein is known to possess pro-angiogenic features, which also contributes to its involvement in the progression of tumorigenesis [51,52,53]. As galectin-1 expression was not changed between unstimulated and cytokine stimulated CardAP-EVs in the previous study, the currently observed differences in the increase of angiogenesis might be due to other transported molecules. Therefore, a broad miRNA screening was performed with the help of an nCounter^®^ miRNA expression assay (NanoString Technologies). Unstimulated CardAP-EVs carried a higher copy number of miRNA302d-3p in comparison to cytokine stimulated CardAP-EVs, which was additionally verified by qPCR. Others have shown that this miRNA stimulated HUVECs to release more VEGF as well as to increase their tube formation capabilities in comparison to untreated controls [41]. Additionally, this group demonstrated that the pro-angiogenic potential of miRNA302-3p was abolished in inhibition experiments using siRNA and other inhibitors of the involved pathway. It is very intriguing to link the amplified release of VEGF by HUVECs treated with unstimulated CardAP-EVs to the enhanced occurrence of transferred miRNA302-3p in exactly that CardAP-EV population. However, it is unlikely that this single miRNA is exclusively responsible for the observed pro- angiogenic effect. Other miRNAs have also been described to be important for the support of angiogenesis, including miRNA 125a, miRNA 214, miRNA 126, miRNA 210, miRNA 132, and miRNA 146a [6,42,43,44]. Interestingly, we also detected miRNA 146a-5p and miRNA 132 to be present in unstimulated as well as cytokine stimulated CardAP-EVs by nCounter^®^ miRNA expression assay as well as by qPCR. Additionally, we detected another miRNA candidate regulating angiogenesis. MiRNA 494 was shown to be enriched in cytokine stimulated CardAP-EVs as determined by nCounter^®^ miRNA expression assay and qPCR. There are contradictory reports on the function of this miRNA, since it has been shown to have both enhancing [45] and inhibitory [46] effects on angiogenesis.

Since miRNAs influence the transcription of proteins, they have to be transported into a cell to be effective. Consequently, their transporting vehicle, in this case the CardAP-EVs, have to be internalized. We have shown that cells internalize CardAP-EVs after 24 h by performing a commonly used method for intracellular staining for cytokines or transcription factors. We incubated CardAP-EVs, originating from a human cell source, with two different murine cardiac cell types and determined the intracellular and extracellular occurrence of human markers. The presence of these markers on CardAP-EVs was verified in advance. The detection of tetraspanins (CD9, CD63, CD81), a characteristic adhesion molecule (CD29) as well as the absence of GM130 would fulfill the requirements for EVs according to the existing guidelines [11,54,55]. The intra-/extracellular staining method was used for the very first time, to our knowledge, in the context of analyzing the EV uptake and interaction with cells. Admittedly, this method cannot elucidate the efficiency of EV uptake and is highly dependent on the present markers on investigated EVs. However, it enables an easy method to determine intracellular uptake of EVs and can complement the current used methods.

Other studies identified the interaction with recipient cells by tracking and determining the signal of fluorescently labelled EVs [56,57,58]. In the present study, we labelled CardAP-EVs with DiD or PKH26 and observed a constant increase of DiD^+^ cells over 24 h by flow cytometry. Interestingly, the interaction was independent of the cell species as murine and human cells showed comparable CardAP-EV interaction. A control for the dye was incorporated in the study, because dyes can form vesicle-like compartments and cause false positive signals [57]. The fluorescence control showed a negligible signal after 24 h incubation with murine or human cells. This emphasizes that the observed shift of fluorescence is due to the interaction of CardAP-EVs with those cells and hence a true positive result. Additionally, we observed PKH26 fluorescently labelled CardAP-EVs in close proximity to host cell nuclei, which is likely caused by an intracellular location of CardAP-EVs that was determined by the intra-/extracellular staining. We speculate that the intracellular uptake of CardAP-EVs and their localization close to the nucleus are important for the function of carried miRNAs.

In conclusion, we have shown that EVs from cardiac cells are able to support angiogenesis. However, differences were discovered between EVs from unstimulated and cytokine stimulated cardiac cells in regard to their miRNA composition, which seems to be connected with the provoked release of different pro-angiogenic factors after their internalization into endothelial cells. This new insight allows now for a selection of potent targets to develop a more efficient and safe therapeutic intervention.

## 4. Materials and Methods

### 4.1. Isolation of Human CardAP Cells

Human CardAP cells were generated by outgrowth cultures of endomyocardial biopsies from the right or left ventricular side of the interventricular septum as previously described [38]. Tissues were obtained according to the local guidelines of the Charité-Universitätsmedizin Berlin and the study was approved on 14th November 2016 by the ethics committee of the Charité-Universitätsmedizin Berlin (No. EA2/140/16).

### 4.2. Cell Culture of Primary Cells and Cell Lines

In general, cryopreserved cells were thawed and cultured in the appropriate media for at least one passage till usage. HL-1 cells were cultured in Claycomb medium (Sigma-Aldrich, St. Louis, MO, USA) supplemented with 10% fetal bovine serum (Biochrom, Berlin, Germany), 1% penicillin/streptomycin, 2 mM glutamine (all provided by Gibco^®^ Life Technologies, Grand Island, NY, USA) and 100 µM norepinephrine (Sigma, Steinheim, Germany). MHEC5-T cells were cultured in DMEM (Biochrom, Berlin, Germany), and supplemented with 1% penicillin/streptomycin, 10% fetal calf serum and 2 mM L-glutamine. For the culture of CardAP cells and human umbilical vein endothelial cells (HUVECs, purchased from Cascade Biologics^®^, Thermo Fisher Scientific, Rochester, NY, USA) the human serum (German Red Cross, Berlin, Germany) was ultracentrifuged (UC serum) to avoid contamination of extracellular vesicles (EVs) by the serum source. Therefore, medium supplemented with 50% human serum was centrifuged for 24 h at 100,000× *g* (L7-55 ultracentrifuge with SW-32 Ti buckets; all from Beckman coulter, Palo Alto, CA, USA). CardAP cells were cultured in IDH medium (equal amounts of IMDM/DMEM/Ham´s F12; all Biochrom) supplemented with 10% UC serum, 1% penicillin/streptomycin, 20 ng/mL basic fibroblast growth factor and 10 ng/mL epithelial growth factor (both from Preprotech, Hamburg, Germany). HUVECs were cultured in EBM medium (Lonza, Walkersville, MD, USA) supplemented with 10% UC serum, 1% penicillin/streptomycin, 2 mM L-glutamine and most supplements of the EGM-2 BulletKit (Lonza, Walkersville, MD, USA). Here, the fetal calf serum was not supplemented, since the UC serum was used for cultivation. Unless stated otherwise, primary cells were used in all in vitro assays from passage 2 to 7.

### 4.3. Isolation and Characterization of CardAP-EVs

CardAP-EVs were isolated by differential centrifugation as recently described [40]. Briefly, conditioned medium was collected from 80% confluent CardAP cells, which had been incubated for 20 h in serumfree IDH medium either in the presence or absence of 10 ng/mL of human TNFα, human IFNγ and human IL-1β (all purchased from Miltenyi Biotec, Bergisch Gladbach, Germany). The conditioned medium then underwent a stepwise centrifugation at 300× *g* for 10 min, at 2000× *g* for 20 min, at 12,000× *g* for 45 min and at 100,000× *g* for 165 min (Allegra^®^ X-15R centrifuge and L7-55 ultracentrifuge with SW-32 Ti buckets; all from Beckman coulter, Brea, CA, USA). The last centrifugation step was repeated to wash the EV pellet with 0.1 µm filtered PBS (Biochrom, Berlin, Germany) For dye labelling CardAP-EVs, EVs were incubated with either 6 µL Vybrant^®^ DiD cell label solution (Invitrogen™, Molecular Probes, Eugene, OR, USA) or 3 µL PKH26 (PKH26 Red Fluorescent Cell Linker Kits for General Cell Membrane Labelling, Sigma Aldrich, St. Louis, MO, USA) in 6 mL PBS for 10 min on ice prior to the final centrifugation step. A negative control for labelling was processed in the same way by labelling unconditioned serumfree IDH medium to exclude false positive staining by DiD or PKH26 aggregates. All samples were reconstituted in 500 µL of 0.1 µm filtered PBS, transferred to low-binding tubes (Sarstedt, Nümbrecht, Germany) and stored at −80 °C till further usage.

All batches of isolated CardAP-EVs were analyzed for their protein content by Pierce™ BCA protein assay (Thermo Scientific, Rockford, IL, USA) and investigated for surface marker by flow cytometry as previously described [40]. In this study, CardAP-EVs coupled to 4 µm aldehyde/sulfate beads (Molecular Probes^®^, Life Technologies, Eugene, OR, USA) were additionally tested for the presence of GM130. Therefore, CardAP-EVs bound to beads were washed in FACS buffer (1% fetal calf serum in PBS) and then stained for 30 min with the anti-GM130/GOLGA2 antibody (Novius Biologicals, Abingdon, UK). Afterwards, the samples were washed, incubated in FACS buffer containing rabbit serum (Sigma AldrichSt. Louis, MO, USA) for 15 min and afterwards stained for 30 min with an anti-rabbit AF488 labelled antibody (Molecular Probes, Eugene, OR, USA). Finally, the samples were washed, fixed in 0.5% PFA supplemented FACS buffer and stored at 4 °C until measurement on a MACSQuant (Miltenyi Biotec, Bergisch Gladbach, Germany). Additionally, staining controls were included for the secondary antibody (anti-rabbit AF488; negative control) alone as well as apoptotic beads bound to aldehyde/sulfate beads (positive control).

### 4.4. Interaction Studies of Labelled CardAP-EVs with Different Recipient Cells

Murine HL-1 or MHEC5-T cells were cultured in a 48-well-plate (2 × 10^5^/well) and treated either with 6 µg DiD labelled CardAP-EVs or the equal volume of DiD negative control. Cells were harvested at different time points (0, 2, 7, 19, and 24 h) for analysis by flow cytometry. Cells were washed twice with PBS and harvested by Accutase (Gibco^®^, Life Technologies) treatment. Afterwards, the cells were labelled in FACS buffer with a dead/viable marker (V510, 1:100; LIVE/DEAD^®^ Fixable Aqua Dead Cell Stain Kit; Invitrogen/Thermo Fisher Scientific, Eugene, OR, USA) for 20 min, washed with FACS buffer and fixed with 0.5% PFA supplemented FACS buffer. All samples were measured at the Canto II (BD Biosciences, Heidelberg, Germany) and all flow cytometry data were analyzed with FlowJo 10.2. Software (FlowJo™, LLC, RO, USA).

Microscopic analysis was performed to visualize the interaction between labelled CardAP-EVs and recipient cells. DiD labelled HL-1 cells were cultured in a 48-well-plate (2 × 10^5^/well) and treated with 6 µg PKH26 labelled CardAP-EVs or the equal volume of the PKH26 negative control. The interaction was stopped at different time points (0, 2, 7, 19, and 24 h). Here, cells were washed with PBS, fixed with 4 % PFA in PBS and afterwards labelled with 4´,6-diamidino-2-phenylindole (DAPI; Molecular Probes^®^, Life Technologies, Waltham. MA, USA). After two washing steps, cells were examined in 20× magnification with the help of an AxioObserver microscope running AxioVision software (both from Carl Zeiss Microscopy GmbH, Jena, Germany).

### 4.5. Combined Intra- and Extracellular Staining to Monitor Interaction of CardAP-EVs with Recipient Cells

The interaction of CardAP-EVs with murine cells was assessed by evaluating human CardAP-EV markers within or on murine cells by an intra- or extracellular staining. HL-1 or MHEC5-T cells were cultured in a 48-well-plate (2 × 10^5^/well) and treated with 6 µg CardAP-EVs either unstimulated or cytokine stimulated, or they were left untreated. Cells were harvested after 24 h by Accutase treatment, evenly distributed into at least two 5 mL Polystyrene Round-Bottom Tubes (Falcon^®^, Corning Science México, Tamaulipas, Mexico) and washed once in FACS buffer. For the extracellular staining (surface staining), both murine cell types were stained for 30 min in the dark with human-specific antibodies for CD73-APC, CD63-PE and CD81-FITC, (all 1:50 and purchased from Biolegend, San Diego, CA, USA) and a dead/viable marker as previously stated. The intracellular staining for the listed human markers was done according to the manual of Foxp3/Transcription Factor Staining Buffer Set (Invitrogen by Thermo Fisher Scientific, Carlsbad, CA, USA). Firstly, cells were stained for the viability marker as stated beforehand. Secondly, cells were permeabilized with the provided Fix/Perm solution. Finally, these cells were stained intracellularly with the same antibodies used for the extracellular stain except the dead/viable marker. Afterwards, labelled cells were washed and fixed with 0.5% PFA until measurement at the Canto II. Controls included untreated cells stained with the anti-human antibodies as well as CardAP-EV treated cells stained with isotope control antibodies (mouse IgG1, kappa APC for CD73 APC, purchased from Miltenyi, mouse IgG1, kappa PE for CD63 PE, mouse IgG1, kappa FITC for CD81 FITC, both purchased from Biolegend).

### 4.6. Matrigel Based Tube Formation Assay

HUVECs were seeded in 6-well plates (1.9 × 10^5^/well) 24 h prior to a Matrigel-based tube formation assay. They were treated with 6 µg/mL of either unstimulated or cytokine stimulated CardAP-EVs, PBS in equal volumes to EVs or were left untreated. The next day, HUVECs were harvested by Accutase treatment and used for Matrigel-based tube formation assays. Pre-cooled 24-well plates were coated with 200 µL Matrigel (Corning) per well. The plates remained on ice until the transfer for 30 min to 37 °C with 5% CO2 to allow the gel to set. Afterwards, each well was washed with PBS before adding 1 × 10^5^ HUVECs in 400 µL EBM medium. A positive control was included by adding 10 ng/mL VEGF (Miltenyi) to untreated HUVECs. Furthermore, each condition was performed in duplicates and the network formation of the tubular structures was documented after 20–24 h with an AxioObserver microscope running AxioVision software (both Carl Zeiss Microscopy, Jena, Germany). Eight random images were taken for each sample and quantified for the tubular network formation (number of junctions and total branching length) by performing analysis with the Angiogenesis Analyzer plugin for the free available software ImageJ1.50i (Wayne Rasband, NIH, USA).

### 4.7. Detection of Pro-Angiogenic Factor Release in HUVEC Cultures

Supernatants of treated HUVECs were analyzed for human VEGF (Quantikine^®^ ELISA, R&D Systems, Biotechne brand, Mineapolis, MN, USA), IL-6 and IL-8 (both ELISA MAX™ Deluxe; Biolegend) using an enzyme-linked immunosorbent assay (ELISA) according to the manufacturer’s protocol. In brief, ELISA microplates (Nunc™ MaxiSorp™ ELISA Plates, Uncoated, Thermo Fisher Scientific, Rochester, NY, USA) were coated overnight with the capture antibody (1:200) and followed by several washing steps using PBS + 0.05% Tween 20 (Sigma Aldrich, St. Louis, MO, USA) on the next day. Microplates were blocked with the blocking buffer for at least 3 h. Next, samples and freshly prepared standards were added to the wells for an overnight incubation. On the next day, the plates were washed several times and the detection antibody (1:200) was applied for additional 2 h. After washing, the avidin-HRP conjugate (1:1000) was added to each well and washed away after 1 h by thorough washing. Fresh TMB substrate was added to the wells and incubated for 15–20 min. The reaction was stopped with stop solution and the absorbance was measured at 450 nm and 570 nm on a plate reader (Mithras LB 940 and MikroWin Version 4.41 software, both from Berthold Technologies, Bad Wildbad, Germany). For normalization to the cell amount, HUVECs were stained with crystal violet (Merck Chemicals, Darmstadt, Germany) and the absorbance was measured at 495 nm on the same plate reader as the ELISAs. Data are represented as the ration of the ELISA results towards the crystal violet determination.

### 4.8. Extraction and Expression Analysis of miRNAs from CardAP-EVs

MiRNA was extracted from CardAP-EVs after the final centrifugation step by using the miRNeasy Mini Kit (Qiagen, Valencia, CA) according to manufacturer’s instructions. The RNA eluate was evaluated for its concentration with the NanoDrop 2000 spectrophotometer (Thermo Fisher Scientific, Waltham, MA, USA). An overview of the miRNA content was achieved by performing an analysis with the nCounter^®^ Analysis System (NanoString Technologies, Seattle, WA, USA). MiRNA isolates from each condition of three different CardAP donors were investigated with the help of the nCounter^®^ Human v2 miRNA expression assay panel, according to the manufacturer’s protocol (NanoString Technologies) at an nCounter^®^ Digital Analyzer. Analysis of the gathered raw data for miRNAs was performed with nSolver software (version 4.0, NanoString Technologies, Seattle, WA USA, http://www.NanoString.com/products/nSolver). Therefore, the data was normalized using the top 100 most abundant miRNAs in all samples as well as the positive controls to normalize for any differences in preparation, hybridization, and processing efficiency. Normalized miRNA counts were used for further analysis and a background correction was used by subtracting the mean + 2 standard derivations of negative control. Afterwards, a count of 10 or more copy numbers was used to define the presence of a given miRNA in CardAP-EVs. Furthermore, at least two of the three donors had to display the miRNA to be considered for further analysis. This normalized data was further investigated for enrichment in biological pathways and in comparative Venn diagram by an open-source software called Functional Enrichment Analysis Tool (FunRich, version 3.1.3, http://www.funrich.org) [59,60].

Additionally, some miRNAs were evaluated by quantitative poly chain reaction (qPCR). Therefore, the 10 ng of RNA was reverse transcribed with TaqMan^®^ Advanced miRNA cDNA Synthesis Kit and used for PCR reaction with TaqMan^®^ Fast Advanced Master Mix, all according the manufacture´s protocol. The qPCR was performed on a QuantStudio 6 Flex Real-Time PCR machine (Applied Biosystems, Thermo Fisher Scientific, Darmstadt, Germany). Three technical replicates of each sample were analyzed for gene expression for the following human miRNAs: 494-3p,146a-5p, 132-3p, 26b-5p, 199a-3p, 186-5p, and 302d-3p (purchased as TaqMan^®^ Advanced miRNA Assay; all from Applied Biosystems, Thermos Fisher). The samples were normalized to the expression of the median of miRNA26b-5p and miRNA199a-3p, which were identified to be the most stable in CardAP-EV preparations by NormFinder Excel Add-In v0.953 [61]. The data was analyzed with the delta-delta CT (∆∆ CT) method. Therefore, the final results were calculated as fold change of target miRNA expression in unstimulated CardAP-EVs relative to the cytokine stimulated CardAP-EV reference samples to demonstrate differentially expressed miRNAs.

### 4.9. Statistical Analysis

All data are shown as median with interquartile range, if not indicated otherwise, and statistical analysis was performed using GraphPad Prism 6.0 software (GraphPad Software Inc, San Diego, CA, USA). First, data were tested with Shapiro-Wilk test for normal distribution. For the comparison of the different groups, data were tested by repeated measures test for One-Way ANOVA. Therefore, parametric data were tested with repeated measures ANOVA (Bonferroni post hoc test) or non-parametric data sets were tested with Friedman test (with Dunn’s post hoc test). Statistical differences between two groups with only one variable in paired observations were determined either for non-parametric samples using with the Wilcoxon matched-pairs signed rank test or for parametric samples with the paired T test. For unpaired non-parametric observations, the data were analyzed with Kruskal-Wallis test (with Dunn’s post hoc test). Results were considered significant with * *p* < 0.05, ** *p* < 0.01, *** *p* < 0.001.

## Figures and Tables

**Table 1:** Involvement of selected miRNAs differently detected between both CardAP-EV populations by nCounter^®^ miRNA expression assay in angiogenesis. RNA of CardAP-EVs was analyzed by nCounter^®^ miRNA expression assay for both EV generation conditions (unstimulated and cytokine stimulated) from three different CardAP donors. The normalized and background subtracted results for the median counts were used to calculate the fold change between both conditions (unstimulated/cytokine stimulated, =1.0 no difference between each other, >1 increased in unstimulated CardAP-EVs, <1 decreased in unstimulated CardAP-EVs, =0.00 just detectable in cytokine stimulated CardAP-EVs). VEGF = vascular endothelial growth factor, HUVECs = human umbilical vein endothelial cells, ECs = endothelial cells, RASA1 = Ras P21 protein activator1, PDGFRA = platelet derived growth factor receptor alpha.

MiRNAs	Fold Change CardAP-EVs to CardAP-EVs^(cyt)^	Involvement in Angiogenesis
302d-3p	3.03	Pro-angiogenic, HUVECs increase VEGF release [41]
186-5p	1.13	Unknown
132-3p	0.61	Pro-angiogenic, suppresses GTPase activating proteins RASA1 [42]
146a-5p	0.39	Pro-angiogenic, ECs increase PDGFRA expression [43] and MSCs increase VEGF release [44]
494-3p	0.00	Contradictory roles, pro-angiogenic [45] as well as anti-angiogenic [46]

## Supplementary Materials

Supplementary materials can be found at https://www.mdpi.com/1422-0067/20/24/6363/s1.

## Abbreviations

IFNγ	Interferon gamma
TNFα	Tumor necrosis factor alpha
IL	Interleukin
CardAP	Human cardiac-derived adherent proliferating
MSC	Mesenchymal stromal cells
HUVECs	Human umbilical vein endothelial cells

## References

[1] Théry C., Ostrowski M., Segura E. (2009). Membrane vesicles as conveyors of immune responses. Nat. Rev. Immunol..

[2] Yoon Y.J., Kim O.Y., Gho Y.S. (2014). Extracellular vesicles as emerging intercellular communicasomes. BMB Rep..

[3] Alfaro M.P., Saraswati S., Young P.P. (2011). Molecular Mediators of Mesenchymal Stem Cell Biology. Vitam. Horm..

[4] Collino F., Bruno S., Deregibus M.C., Tetta C., Camussi G. (2011). MicroRNAs and Mesenchymal Stem Cells. Vitam. Horm..

[5] Loewenstein W.R. (1981). Junctional intercellular communication: the cell-to-cell membrane channel. Physiol. Rev..

[6] Emanueli C., Shearn A.I.U., Angelini G.D., Sahoo S. (2015). Exosomes and exosomal miRNAs in cardiovascular protection and repair. Vascul. Pharmacol..

[7] Hynes B., Kumar A.H.S., O’Sullivan J., Klein Buneker C., Leblond A.-L., Weiss S., Schmeckpeper J., Martin K., Caplice N.M. (2013). Potent endothelial progenitor cell-conditioned media-related anti-apoptotic, cardiotrophic, and pro-angiogenic effects post-myocardial infarction are mediated by insulin-like growth factor-1. Eur. Heart J..

[8] Menter T., Tzankov A. (2018). Mechanisms of Immune Evasion and Immune Modulation by Lymphoma Cells. Front. Oncol..

[9] Becker A., Thakur B.K., Weiss J.M., Kim H.S., Peinado H., Lyden D. (2016). Extracellular Vesicles in Cancer: Cell-to-Cell Mediators of Metastasis. Cancer Cell.

[10] Kowal J., Arras G., Colombo M., Jouve M., Morath J.P., Primdal-Bengtson B., Dingli F., Loew D., Tkach M., Théry C. (2016). Proteomic comparison defines novel markers to characterize heterogeneous populations of extracellular vesicle subtypes. Proc. Natl. Acad. Sci..

[11] Witwer K.W., Buzás E.I., Bemis L.T., Bora A., Lässer C., Lötvall J., Nolte-’t Hoen E.N., Piper M.G., Sivaraman S., Skog J. (2013). Standardization of sample collection, isolation and analysis methods in extracellular vesicle research. J. Extracell. Vesicles.

[12] Andreu Z., Yáñez-Mó M. (2014). Tetraspanins in extracellular vesicle formation and function. Front. Immunol..

[13] Sluijter J.P.G., Verhage V., Deddens J.C., Van Den Akker F., Doevendans P.A. (2014). Microvesicles and exosomes for intracardiac communication. Cardiovasc. Res..

[14] Kowal J., Tkach M., Théry C. (2014). Biogenesis and secretion of exosomes. Curr. Opin. Cell Biol..

[15] Lener T., Gimona M., Aigner L., Börger V., Buzas E., Camussi G., Chaput N., Chatterjee D., Court F.A., del Portillo H.A. (2015). Applying extracellular vesicles based therapeutics in clinical trials - An ISEV position paper. J. Extracell. Vesicles.

[16] Park K.S., Bandeira E., Shelke G.V., Lässer C., Lötvall J. (2019). Enhancement of therapeutic potential of mesenchymal stem cell-derived extracellular vesicles. Stem Cell Res. Ther..

[17] Fleury A., Martinez M.C., Le Lay S. (2014). Extracellular vesicles as therapeutic tools in cardiovascular diseases. Front. Immunol..

[18] Xiao J., Pan Y., Li X.H., Yang X.Y., Feng Y.L., Tan H.H., Jiang L., Feng J., Yu X.Y. (2016). Cardiac progenitor cell-derived exosomes prevent cardiomyocytes apoptosis through exosomal miR-21 by targeting PDCD4. Cell Death Dis..

[19] Barile L., Milano G., Vassalli G. (2017). Beneficial effects of exosomes secreted by cardiac-derived progenitor cells and other cell types in myocardial ischemia. Stem Cell Investig..

[20] El Harane N., Kervadec A., Bellamy V., Pidial L., Neametalla H.J., Perier M.-C., Lima Correa B., Thiébault L., Cagnard N., Duché A. (2018). Acellular therapeutic approach for heart failure: in vitro production of extracellular vesicles from human cardiovascular progenitors. Eur. Heart J..

[21] Bruno S., Collino F., Iavello A., Camussi G. (2014). Effects of mesenchymal stromal cell-derived extracellular vesicles on tumor growth. Front. Immunol..

[22] Bian S., Zhang L., Duan L., Wang X., Min Y., Yu H. (2014). Extracellular vesicles derived from human bone marrow mesenchymal stem cells promote angiogenesis in a rat myocardial infarction model. J. Mol. Med..

[23] Lombardo G., Dentelli P., Togliatto G., Rosso A., Gili M., Gallo S., Deregibus M.C., Camussi G., Brizzi M.F. (2016). Activated Stat5 trafficking Via Endothelial Cell-derived Extracellular Vesicles Controls IL-3 Pro-angiogenic Paracrine Action. Sci. Rep..

[24] Taraboletti G., D’Ascenzo S., Borsotti P., Giavazzi R., Pavan A., Dolo V. (2002). Shedding of the Matrix Metalloproteinases MMP-2, MMP-9, and MT1-MMP as Membrane Vesicle-Associated Components by Endothelial Cells. Am. J. Pathol..

[25] Barile L., Lionetti V., Cervio E., Matteucci M., Gherghiceanu M., Popescu L.M., Torre T., Siclari F., Moccetti T., Vassalli G. (2014). Extracellular vesicles fromhuman cardiac progenitor cells inhibit cardiomyocyte apoptosis and improve cardiac function aftermyocardial infarction. Cardiovasc. Res..

[26] Wang Y., Dong L., Zhong H., Yang L., Li Q., Su C., Gu W., Qian Y. (2019). Extracellular Vesicles (EVs) from Lung Adenocarcinoma Cells Promote Human Umbilical Vein Endothelial Cell (HUVEC) Angiogenesis through Yes Kinase-associated Protein (YAP) Transport. Int. J. Biol. Sci..

[27] Todorova D., Simoncini S., Lacroix R., Sabatier F., Dignat-George F. (2017). Extracellular vesicles in angiogenesis. Circ. Res..

[28] Zhang H.-C., Liu X.-B., Huang S., Bi X.-Y., Wang H.-X., Xie L.-X., Wang Y.-Q., Cao X.-F., Lv J., Xiao F.-J. (2012). Microvesicles Derived from Human Umbilical Cord Mesenchymal Stem Cells Stimulated by Hypoxia Promote Angiogenesis Both In Vitro and In Vivo. Stem Cells Dev..

[29] Ferreira J.R., Teixeira G.Q., Santos S.G., Barbosa M.A., Almeida-Porada G., Gonçalves R.M. (2018). Mesenchymal Stromal Cell Secretome: Influencing Therapeutic Potential by Cellular Pre-conditioning. Front. Immunol..

[30] Balbi C., Piccoli M., Barile L., Papait A., Armirotti A., Principi E., Reverberi D., Pascucci L., Becherini P., Varesio L. (2017). First Characterization of Human Amniotic Fluid Stem Cell Extracellular Vesicles as a Powerful Paracrine Tool Endowed with Regenerative Potential. Stem Cells Transl. Med..

[31] Almeria C., Weiss R., Roy M., Tripisciano C., Kasper C., Weber V., Egger D. (2019). Hypoxia Conditioned Mesenchymal Stem Cell-Derived Extracellular Vesicles Induce Increased Vascular Tube Formation in vitro. Front. Bioeng. Biotechnol..

[32] Harting M.T., Srivastava A.K., Zhaorigetu S., Bair H., Prabhakara K.S., Toledano Furman N.E., Vykoukal J.V., Ruppert K.A., Cox C.S., Olson S.D. (2018). Inflammation-Stimulated Mesenchymal Stromal Cell-Derived Extracellular Vesicles Attenuate Inflammation. Stem Cells.

[33] Cosenza S., Toupet K., Maumus M., Luz-Crawford P., Blanc-Brude O., Jorgensen C., Noël D. (2018). Mesenchymal stem cells-derived exosomes are more immunosuppressive than microparticles in inflammatory arthritis. Theranostics.

[34] Huang C., Luo W.-F., Ye Y.-F., Lin L., Wang Z., Luo M.-H., Song Q.-D., He X.-P., Chen H.-W., Kong Y. (2019). Characterization of inflammatory factor-induced changes in mesenchymal stem cell exosomes and sequencing analysis of exosomal microRNAs. World J. Stem Cells.

[35] Haag M., Ritterhoff J., Dimura A., Miteva K., Van Linthout S., Tschöpe C., Ringe J., Sittinger M. (2013). Pro-Angiogenic Effect of Endomyocardial Biopsy-Derived Cells for Cardiac Regeneration. Curr. Tissue Eng..

[36] Miteva K., Haag M., Peng J., Savvatis K., Becher P.M., Seifert M., Warstat K., Westermann D., Ringe J., Sittinger M. (2011). Human cardiac-derived adherent proliferating cells reduce murine acute coxsackievirus B3-induced myocarditis. PLoS ONE.

[37] Miteva K., Van Linthout S., Pappritz K., Müller I., Spillmann F., Haag M., Stachelscheid H., Ringe J., Sittinger M., Tschöpe C. (2016). Human Endomyocardial Biopsy Specimen-Derived Stromal Cells Modulate Angiotensin II-Induced Cardiac Remodeling. Stem Cells Transl. Med..

[38] Haag M., Van Linthout S., Schröder S.E.A., Freymann U., Ringe J., Tschöpe C., Sittinger M. (2010). Endomyocardial biopsy derived adherent proliferating cells - A potential cell source for cardiac tissue engineering. J. Cell. Biochem..

[39] Haag M., Stolk M., Ringe J., Van Linthout S., Tschöpe C., Sittinger M., Seifert M. (2013). Immune attributes of cardiac-derived adherent proliferating (CAP) cells in cardiac therapy. J. Tissue Eng. Regen. Med..

[40] Beez C.M., Haag M., Klein O., Van Linthout S., Sittinger M., Seifert M. (2019). Extracellular vesicles from regenerative human cardiac cells act as potent immune modulators by priming monocytes. J. Nanobiotechnol..

[41] Jiang C., Xie P., Sun R., Sun X., Liu G., Ding S., Zhu M., Yan B., Liu Q., Chen X. (2018). c-Jun-mediated microRNA-302d-3p induces RPE dedifferentiation by targeting p21Waf1/Cip1. Cell Death Dis..

[42] Lei Z., van Mil A., Brandt M.M., Grundmann S., Hoefer I., Smits M., El Azzouzi H., Fukao T., Cheng C., Doevendans P.A. (2015). MicroRNA-132/212 family enhances arteriogenesis after hindlimb ischaemia through modulation of the Ras-MAPK pathway. J. Cell. Mol. Med..

[43] Zhu K., Pan Q., Zhang X., Kong L.-Q., Fan J., Dai Z., Wang L., Yang X.-R., Hu J., Wan J.-L. (2013). MiR-146a enhances angiogenic activity of endothelial cells in hepatocellular carcinoma by promoting PDGFRA expression. Carcinogenesis.

[44] Seo H.-H., Lee S.-Y., Lee C.Y., Kim R., Kim P., Oh S., Lee H., Lee M.Y., Kim J., Kim L.K. (2017). Exogenous miRNA-146a Enhances the Therapeutic Efficacy of Human Mesenchymal Stem Cells by Increasing Vascular Endothelial Growth Factor Secretion in the Ischemia/Reperfusion-Injured Heart. J. Vasc. Res..

[45] Mao G., Liu Y., Fang X., Liu Y., Fang L., Lin L., Liu X., Wang N. (2015). Tumor-derived microRNA-494 promotes angiogenesis in non-small cell lung cancer. Angiogenesis.

[46] Chen S., Zhao G., Miao H., Tang R., Song Y., Hu Y., Wang Z., Hou Y. (2015). MicroRNA-494 inhibits the growth and angiogenesis-regulating potential of mesenchymal stem cells. FEBS Lett..

[47] Franzin C., Ulivi V., Pascucci L., Bosco M.C., Pozzobon M., Principi E., Varesio L., Becherini P., Reverberi D., Balbi C. (2017). Mesenchymal Stem Cell-Derived Extracellular Vesicles as Mediators of Anti-Inflammatory Effects: Endorsement of Macrophage Polarization. Stem Cells Transl. Med..

[48] Li A., Dubey S., Varney M.L., Dave B.J., Singh R.K. (2003). IL-8 directly enhanced endothelial cell survival, proliferation, and matrix metalloproteinases production and regulated angiogenesis. J. Immunol..

[49] Cohen T., Nahari D., Cerem L.W., Neufeld G., Levi B.-Z. (1996). Interleukin 6 Induces the Expression of Vascular Endothelial Growth Factor. J. Biol. Chem..

[50] Anderson J.D., Johansson H.J., Graham C.S., Vesterlund M., Pham M.T., Bramlett C.S., Montgomery E.N., Mellema M.S., Bardini R.L., Contreras Z. (2016). Comprehensive proteomic analysis of mesenchymal stem cell exosomes reveals modulation of angiogenesis via nuclear factor-kappaB signaling. Stem Cells.

[51] Thijssen V.L., Griffioen A.W. (2014). Galectin-1 and -9 in angiogenesis: A sweet couple. Glycobiology.

[52] Thijssen V.L.J.L., Postel R., Brandwijk R.J.M.G.E., Dings R.P.M., Nesmelova I., Satijn S., Verhofstad N., Nakabeppu Y., Baum L.G., Bakkers J. (2006). Galectin-1 is essential in tumor angiogenesis and is a target for antiangiogenesis therapy. Proc. Natl. Acad. Sci. USA.

[53] Tang D., Gao J., Wang S., Ye N., Chong Y., Huang Y., Wang J., Li B., Yin W., Wang D. (2016). Cancer-associated fibroblasts promote angiogenesis in gastric cancer through galectin-1 expression. Tumor Biol..

[54] Théry C., Clayton A., Amigorena S., Raposo G. (2006). Isolation and Characterization of Exosomes from Cell Culture Supernatants. Current Protocols in Cell Biology.

[55] Lötvall J., Hill A.F., Hochberg F., Buzas E.I., Vizio D.D., Gardiner C., Gho Y.S., Kurochkin I.V., Mathivanan S., Quesenberry P. (2014). Minimal experimental requirements for definition of extracellular vesicles and their functions: A position statement from the International Society for Extracellular Vesicles. J. Extracell. Vesicles.

[56] Chen L., Wang Y., Pan Y., Zhang L., Shen C., Qin G., Ashraf M., Weintraub N., Ma G., Tang Y. (2013). Cardiac progenitor-derived exosomes protect ischemic myocardium from acute ischemia/reperfusion injury. Biochem. Biophys. Res. Commun..

[57] Dominkuš P.P., Stenovec M., Sitar S., Lasič E., Zorec R., Plemenitaš A., Žagar E., Kreft M., Lenassi M. (2018). PKH26 labeling of extracellular vesicles: Characterization and cellular internalization of contaminating PKH26 nanoparticles. Biochim. Biophys. Acta-Biomembr..

[58] Matula Z., Németh A., Lőrincz P., Szepesi Á., Brózik A., Buzás E.I., Lőw P., Német K., Uher F., Urbán V.S. (2016). The Role of Extracellular Vesicle and Tunneling Nanotube-Mediated Intercellular Cross-Talk Between Mesenchymal Stem Cells and Human Peripheral T Cells. Stem Cells Dev..

[59] Pathan M., Keerthikumar S., Chisanga D., Alessandro R., Ang C.-S., Askenase P., Batagov A.O., Benito-Martin A., Camussi G., Clayton A. (2017). A novel community driven software for functional enrichment analysis of extracellular vesicles data. J. Extracell. Vesicles.

[60] Pathan M., Keerthikumar S., Ang C.-S., Gangoda L., Quek C.Y.J., Williamson N.A., Mouradov D., Sieber O.M., Simpson R.J., Salim A. (2015). FunRich: An open access standalone functional enrichment and interaction network analysis tool. Proteomics.

[61] Andersen C.L., Jensen J.L., Ørntoft T.F. (2004). Normalization of Real-Time Quantitative Reverse Transcription-PCR Data: A Model-Based Variance Estimation Approach to Identify Genes Suited for Normalization, Applied to Bladder and Colon Cancer Data Sets. Cancer Res..

