# Supplementary material for: Cardiac Extracellular Vesicles (EVs) Released in the Presence or Absence of Inflammatory Cues Support Angiogenesis in Different Manners
Source: Int J Mol Sci. 2019 Dec 17;20(24):6363. doi: 10.3390/ijms20246363 (PMC6940836; doi:10.3390/ijms20246363)
Supplement: Supplementary file 1 [file ijms-20-06363-s001.pdf]

International Journal of
Molecular Sciences

Supplementary material

Cardiac Extracellular Vesicles (EVs) Released in the Presence or Absence of Inflammatory Cues Support Angiogenesis in Different Manners

Christien Madlen Beez [1,2], Maria Schneider [1,2], Marion Haag [1,3], Kathleen Pappritz [1,4,5], Sophie Van Linthout [1,4,5], Michael Sittinger [1,3] and Martina Seifert [1,2,*]

1 Charité-Universitätsmedizin Berlin, BCRT - Berlin Institute of Health (BIH) Centre for Regenerative Therapies, 10178 Berlin, Germany
2 Institute of Medical Immunology, Charité-Universitätsmedizin Berlin, corporate member of Freie Universität Berlin, Humboldt-Universität zu Berlin, and Berlin Institute of Health, Berlin 13353, Germany
3 Tissue Engineering Laboratory, Charité-Universitätsmedizin Berlin, corporate member of Freie Universität Berlin, Humboldt-Universität zu Berlin, and Berlin Institute of Health, Berlin 13353, Germany
4 Department of Internal Medicine and Cardiology, Campus Virchow Klinikum, Charité-Universitätsmedizin Berlin, corporate member of Freie Universität Berlin, Humboldt-Universität zu Berlin, and Berlin Institute of Health, Berlin 13353, Germany
5 DZHK (German Center for Cardiovascular Research), partner site Berlin, Berlin 10115, Germany

1.1. Supplementary Figures

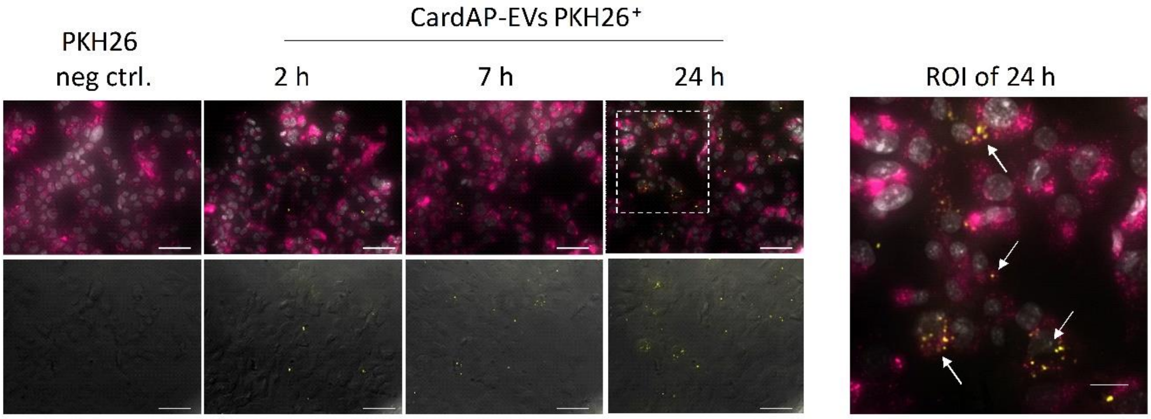

Supplementary Figure S1. CardAP-EVs (EVs from cardiac derived adherent proliferating cells) are interacting with murine cardiomyocytes (HL-1). CardAP-EVs and unconditioned medium (PKH26 negative control) were labelled with PKH26 during the isolation process and applied to cultured HL-1 cells (2×10^5 cells/well). HL-1 cells were labelled with DiD prior to cultivation. At different time points (2, 7 and 24 h) cells were washed with PBS, fixed with 4 % PFA for 30 min and stained with DAPI. Representative pictures of the fluorescence microscopic analysis (*n* = 3, 2 CardAP donors) are shown for each time point as overlay of HL-1 cells (DiD$^+$, pink), nucleus (DAPI, grey pseudo-coloured) und CardAP-EVs (PKH26$^+$, yellow) in the upper raw or as overlay of the bright field image with CardAP-EVs in the lower raw. Scale bars represent 50 µm. In a region of interest (ROI, dotted rectangle in 24 h) an accumulation of CardAP-EVs PKH26$^+$ (ROI of 24 h; white arrows) is observed. Scale bar represents 20 µm.

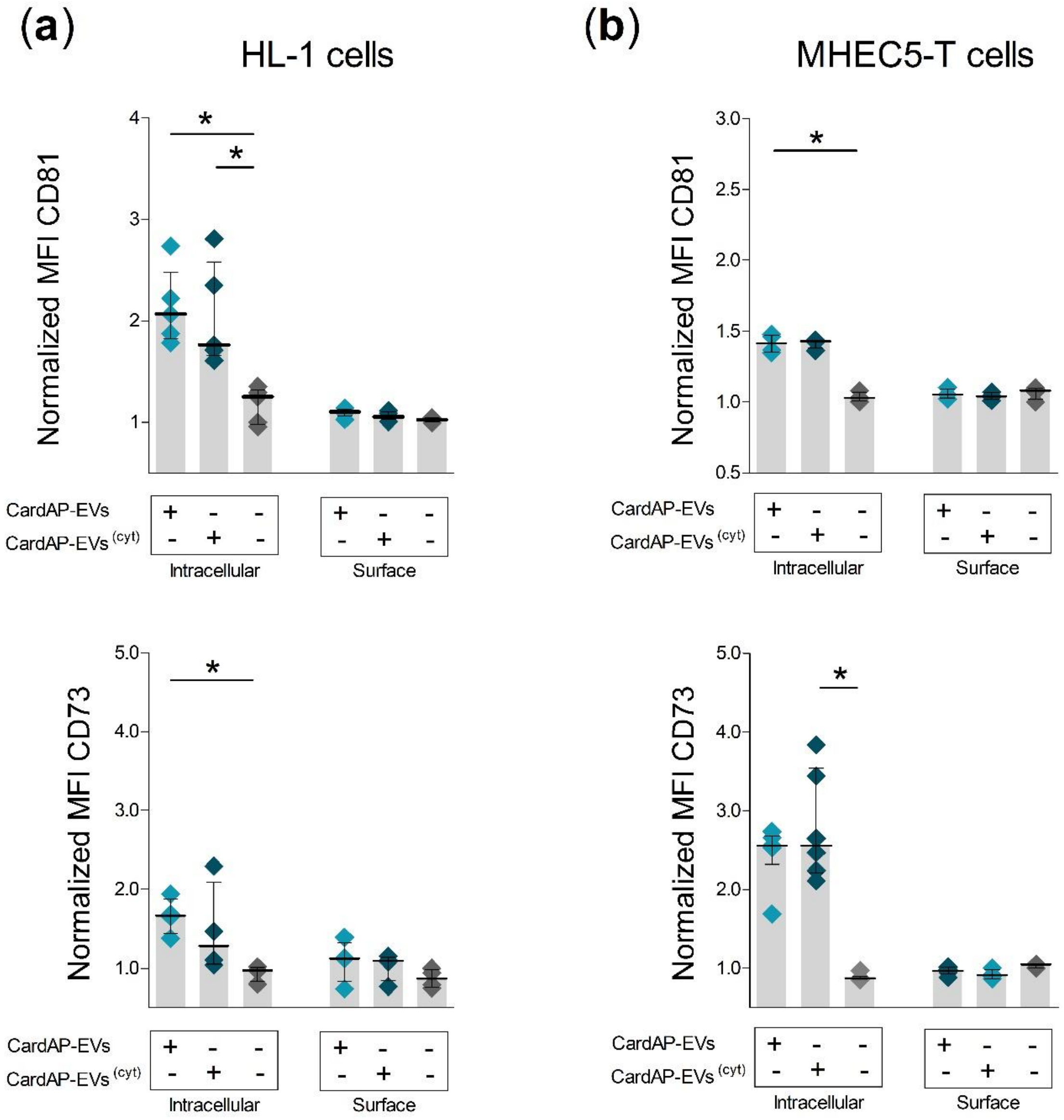

Supplementary Figure S2. CardAP-EVs are internalized by cardiac murine cells. Human CardAP-EVs either unstimulated (CardAP-EVs) or cytokine stimulated (CardAP-EVs$^{(cyt)}$) were applied to cultured murine HL-1 cells or MHEC5-T cells (2×10^5 cells/well). Additionally, an untreated murine cell control was included. After 24 hours, these cells were harvested and stained with anti-human fluorescently labelled antibodies for CD81 or CD73, using either an intracellular or a cell surface staining protocol.Samples were measured by flow cytometry at the CantoII. (**a**), (**b**): The mean fluorescent intensity (MFI) for human CD81 (upper row) and CD73 (lower row) was normalized to an unstained control (intracellular or surface staining) and the normalized MFI is shown as median with interquartile range for both staining conditions as well as different treatments for HL-1 cells (**a**, n = 4–5, 3 different CardAP donors) and MHEC5-T cells (**b**, n= 5, 3 different CardAP donors). Statistical analysis was performed by Kruskal-Wallis test (with Dunns post hoc test, * $p < 0.05$).

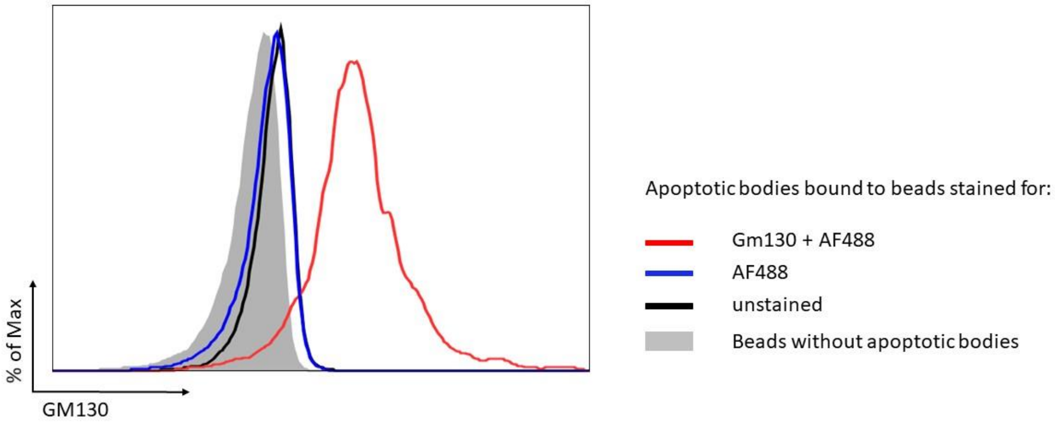

Supplementary Figure S3. GM130 is detectable on apoptotic bodies. During the isolation process a pellet of the apoptotic body fraction (2000× *g* centrifugation step) was collected and applied in the same manner as CardAP-EVs for investigation of GM130 by flow cytometry. The apoptotic boddies were bound to latex/sulfate beads, stained with the anti-GM130 antibody. Before the secondary antibody anti-rabbit AF488 was applied, a blocking step with rabbit serum containg FACS buffer was performed. Finally, the samples were washed, fixed in 0.5% PFA-supplemented FACS buffer and measured at MACSQuant. GM130 was detected for apoptotic bodies when staining was performed with primary and secondary antibody (red line), while no unspecific binding was detected for the staining with secondary antiboby (blue line) as compared to the unstained control (black line).
